# Analysis of heart rate variability and its influencing factors in different blood pressure groups in Changchun City

**DOI:** 10.3389/fpubh.2025.1630953

**Published:** 2025-12-15

**Authors:** YuHang Guo, Jian Li, Feng Guo, HaoRan Sun, YiJin Ning, XinYe Wang

**Affiliations:** 1 School of Clinical Medicine, Changchun University of Chinese Medicine, Changchun, China; 2 Office of the Ethics Committee, Affiliated Hospital of Changchun University of Chinese Medicine, Changchun, China; 3 School of Management, Changchun University of Chinese Medicine, Changchun, China

**Keywords:** heart rate variability, blood pressure population, autonomic nervous system function, cardiovascular event risk, health education and health promotion, disease prevention

## Abstract

**Objective:**

To evaluate the Heart rate variability (HRV) in different blood pressure populations, analyze its influencing factors, rank their importance, and propose targeted health strategies.

**Methods:**

A total of 240 individuals with different blood pressure levels were recruited from a group in Changchun City, Jilin Province, using cluster sampling. Heart rate variability was assessed using the DHD-6000 HRV detector, and influencing factors were evaluated through questionnaires such as the “Health Risk Assessment Questionnaire,” Pittsburgh Sleep Quality Index (PSQI), Subjective Cognitive Decline Questionnaire (SCD-Q9), Generalized Anxiety Disorder 7-item scale (GAD-7), and Patient Health Questionnaire-9 (PHQ-9). Additionally, medical electronic sphygmomanometers (YXY-6) were used to collect blood pressure values. The study comprehensively evaluated Heart rate variability and its influencing factors in different blood pressure populations.

**Results:**

Among the different blood pressure populations in Changchun City, 12 individuals had hypotension (5.0%), 121 had normal blood pressure (50.4%), 87 had prehypertension (36.3%), and 20 had hypertension (8.3%). The detection rate of low TP was 30.4%, indicating weakened autonomic nervous system regulation in this population. Random forest model variable importance ranking showed that hyperlipidemia had the most significant impact on this population. Multivariate linear regression analysis revealed that blood pressure, lipids, marital status, alcohol consumption, age, and exercise had statistically significant effects on Heart rate variability (*p* < 0.05).

**Conclusion:**

Heart rate variability is influenced by multiple factors including lifestyle, blood pressure, lipids, age, and marital status. Among these, hyperlipidemia has the greatest impact on Heart rate variability. Compared to individuals with normal blood pressure, those with prehypertension and hypertension exhibit reduced autonomic nervous system activity and regulatory function. It is recommended to develop reasonable exercise plans, improve unhealthy lifestyle behaviors, control blood pressure and lipids, enhance autonomic nervous system function, and reduce the risk of cardiovascular events.

## 1 Introduction

With the development of the socio-economic environment, improvements in living standards, and changes in lifestyle, the risk of cardiovascular events has increased, and the incidence rate is showing a trend toward younger populations. The activity and regulatory capacity of the autonomic nervous system are significantly related to the occurrence of cardiovascular events. Autonomic imbalance is an important factor in the occurrence of cardiovascular events and is also a direct manifestation of unhealthy behavioral lifestyles and psychological issues. A commonly used quantitative indicator to assess autonomic nervous activity is heart rate variability (HRV) (1–5). Research has found (6) that the measured values of HRV in hypertensive patients are relatively lower compared to healthy populations. The autonomic nervous system is affected, and the physical health condition is gradually deteriorating. This study focuses on analyzing the factors affecting HRV, conducting a comprehensive analysis of HRV levels and influencing factors in different blood pressure groups, and proposing targeted health management strategies.

## 2 Objects and methods

### 2.1 Survey participants

This study is a cross-sectional survey conducted in Changchun, Jilin Province, China, from January to May 2025. The participants were drawn from a resident health-management cohort established by a community health service center in an urban district of Changchun. A cluster-sampling method was used, with the following steps: first, five resident grids were randomly selected from all the grids managed by the community health service center. Then, all permanent residents within these five selected grids who met the inclusion criteria were defined as the survey “clusters.” We contacted every target resident in these grids through the community health-record system and invited them to take part in the survey. Considering an anticipated non-response rate of about 20% for a cross-sectional study, we initially set a target sample size of 300 participants. Ultimately, during the survey period, 260 individuals expressed willingness to participate; after data quality control, the final effective sample size for analysis was 240. According to the “Chinese Guidelines for the Prevention and Treatment of Hypertension (2018 revised edition),” all participants were required to rest in a quiet environment for at least 5 min, then have their seated blood pressure measured three times with a calibrated electronic sphygmomanometer, and the average of the three readings was used for group classification. Inclusion Criteria: ① Age ≥ 18 years; ② Consciousness clear, without cognitive impairment; ③ Adherence to the principle of informed consent, voluntary participation in this survey. Exclusion Criteria: ① Those with severe mental or cognitive impairments; ② Deafness or intellectual disabilities that prevent smooth communication; ③ Participants who dropped out of the study; ④ Those with mental illnesses that prevent self-evaluation; ⑤ Those with severe arrhythmias.

This study has been approved by the Ethics Committee of the Affiliated Hospital of Changchun University of Traditional Chinese Medicine (Approval number CCYFYLL-SQ-2024). We confirm that this research was conducted in accordance with the Declaration of Helsinki (1964) and its subsequent amendments. Before participating in this study, all participants signed an informed consent form. The survey began on January 3, 2025, and ended on May 3, 2025. Non-invasive cardiovascular diagnostic techniques were used, which did not cause physical harm to the participants. Participants’ privacy was strictly protected, and their data were anonymized. This study did not involve minors.

### 2.2 Survey tools

① The “Health Risk Assessment Questionnaire” is used to investigate sociodemographic and behavioral lifestyle factors, including gender, age, marital status, educational level, monthly family income, smoking, alcohol consumption, dietary preferences, water drinking habits, and exercise frequency (7).

② The internationally recognized Pittsburgh Sleep Quality Index (PSQI) is applied to assess sleep quality. The total score ranges from 0 to 21. A PSQI score greater than 7 indicates poor sleep quality, while a score of 7 or less indicates good sleep quality (8). The PSQI has a test–retest reliability of 0.994, a split-half reliability coefficient of 0.824, and a Cronbach’s *α* coefficient of 0.842. Additionally, the structural validity of the PSQI is 0.76, and the classification validity is 0.81, indicating good reliability and validity (9).

③ The application of the Subjective Cognitive Decline Questionnaire-9 (SCD-Q9) indicates that a score of ≥5 suggests potential memory impairment. The Chinese version of the SCD-Q9 has a Cronbach’s *α* coefficient of 0.886 and a split-half reliability coefficient above 0.800, indicating good internal consistency (10).

④ The GAD-7 (Generalized Anxiety Disorder-7) scale is a brief self-report measure designed to assess the presence and severity of generalized anxiety disorder (GAD). According to the evidence provided, a score of ≥5 on the GAD-7 indicates the presence of an anxiety state. The Cronbach’s *α* coefficient for the GAD-7 is 0.867, which suggests good internal consistency reliability. Additionally, the test–retest reliability coefficient is 0.823, indicating that the GAD-7 has good reliability and validity (11).

⑤ The PHQ-9 is a self-administered questionnaire used to assess the severity of depressive symptoms. If the score on the PHQ-9 is ≥5, it indicates the presence of depressive symptoms. The internal consistency coefficient of the PHQ-9, Cronbach’s *α*, is 0.8325, and the test–retest reliability coefficient is 0.932. The sensitivity of the PHQ-9 is 88%, and the specificity is 99%, indicating good reliability and validity (12).

⑥ The DHD-6000 heart rate variability (HRV) detector is used to measure the heart rate variability of subjects. HRV frequency domain analysis can accurately and systematically evaluate the autonomic nervous function comprehensively (13). The significance of common frequency domain indicators is shown in Table 1. Since the actual values of HRV frequency domain indicators do not follow a normal distribution, logarithmic transformation is applied to each HRV frequency domain indicator to achieve a normal distribution before statistical analysis. Normal reference values for short-term HRV frequency domain analysis: TP (6.7 ~ 8.1 ln(ms^2^)), LF (4.7 ~ 7.0 ln(ms^2^)), HF (3.5 ~ 6.8 ln(ms^2^)) (14).

**Table 1:** Commonly used frequency domain indicators in HRV analysis.

Frequency domain indicator	Description	Unit	Meaning
TP	Total power	ms^2^	Reflects the overall activity of the autonomic nervous system
LF	Low frequency Power	ms^2^	Closely related to blood pressure changes, mainly reflects the tension of the sympathetic and parasympathetic nervous systems
HF	High frequency power	ms^2^	Related to respiratory arrhythmia, mainly reflects the tension of the parasympathetic nervous system

⑦ The YXY-6 medical electronic blood pressure monitor is used to measure the blood pressure levels of subjects. According to the hypertension prevention and treatment guidelines in China, blood pressure is classified into three categories: normal blood pressure (systolic pressure <120 mmHg and diastolic pressure <80 mmHg), Prehypertension (systolic pressure 120–139 mmHg and/or diastolic pressure 80–89 mmHg), and hypertension (systolic pressure ≥140 mmHg and/or diastolic pressure ≥90 mmHg). This classification is applicable to adults aged 18 years and older (15).

### 2.3 Statistical methods

The database was established using Epidata 3.1 software, independent sample T-tests and one-way ANOVA were conducted using SPSS 26.0 software, related variables were visualized using GraphPad Prism 8.0 software, Random forest model analysis was performed using R 4.3.3 software, variable selection was conducted using LASSO regression, and the selected variables were included in multiple linear regression analysis with a significance level of *α* = 0.05.

### 2.4 Quality control

Data on different blood pressure groups and heart rate variability were collected to fully understand the background of this study. Surveyors underwent professional training, were clear about the matters that needed attention during the survey process, strictly controlled the use of instruments according to taboos, patiently explained ambiguous questions, and corrected any issues found promptly. After the survey, the surveyors reviewed all questionnaires twice, eliminated invalid ones, checked for any issues, and signed off to ensure the completeness and validity of the survey questionnaires.

## 3 Results

### 3.1 Comparison of HRV frequency domain indicators among different blood pressure characteristics

In this population, there were 12 individuals with low blood pressure, accounting for 5%; 121 individuals with normal blood pressure, accounting for 50.4%; 87 individuals with Prehypertension, accounting for 36.3%; and 20 individuals with hypertension, accounting for 8.3%. The detection rate of low total power (TP) was 30.4%, indicating that the overall autonomic nervous system regulation ability of this population is poor; the detection rate of low frequency power (LF) was 15%, suggesting weakened sympathetic nerve activity; the detection rate of high LF was 20.8%, indicating excessive sympathetic nerve activity. The detection rate of high frequency power (HF) was 35%, excessive parasympathetic activity, which is related to the physiological demands of the body during relaxation and recovery, suggests a tendency toward symptoms such as depression, fatigue, bradycardia, and lowered blood pressure (14), as shown in Table 2.

**Table 2:** Characteristics of blood pressure and HRV frequency domain indicators in different blood pressure populations (*n*%).

Item	Feature	*n*(%)
Blood Pressure	Low blood pressure	12(5.0)
	Normal blood pressure	121(50.4)
	Prehypertension	87(36.3)
	High blood pressure	20(8.3)
TP	Low	73(30.4)
	Normal	108(45.0)
	High	59(24.6)
LF	Low	36(15.0)
	Normal	154(64.2)
	High	50(20.8)
HF	Low	9(3.8)
	Normal	147(61.3)
	High	84(35.0)

**Figure 1:**
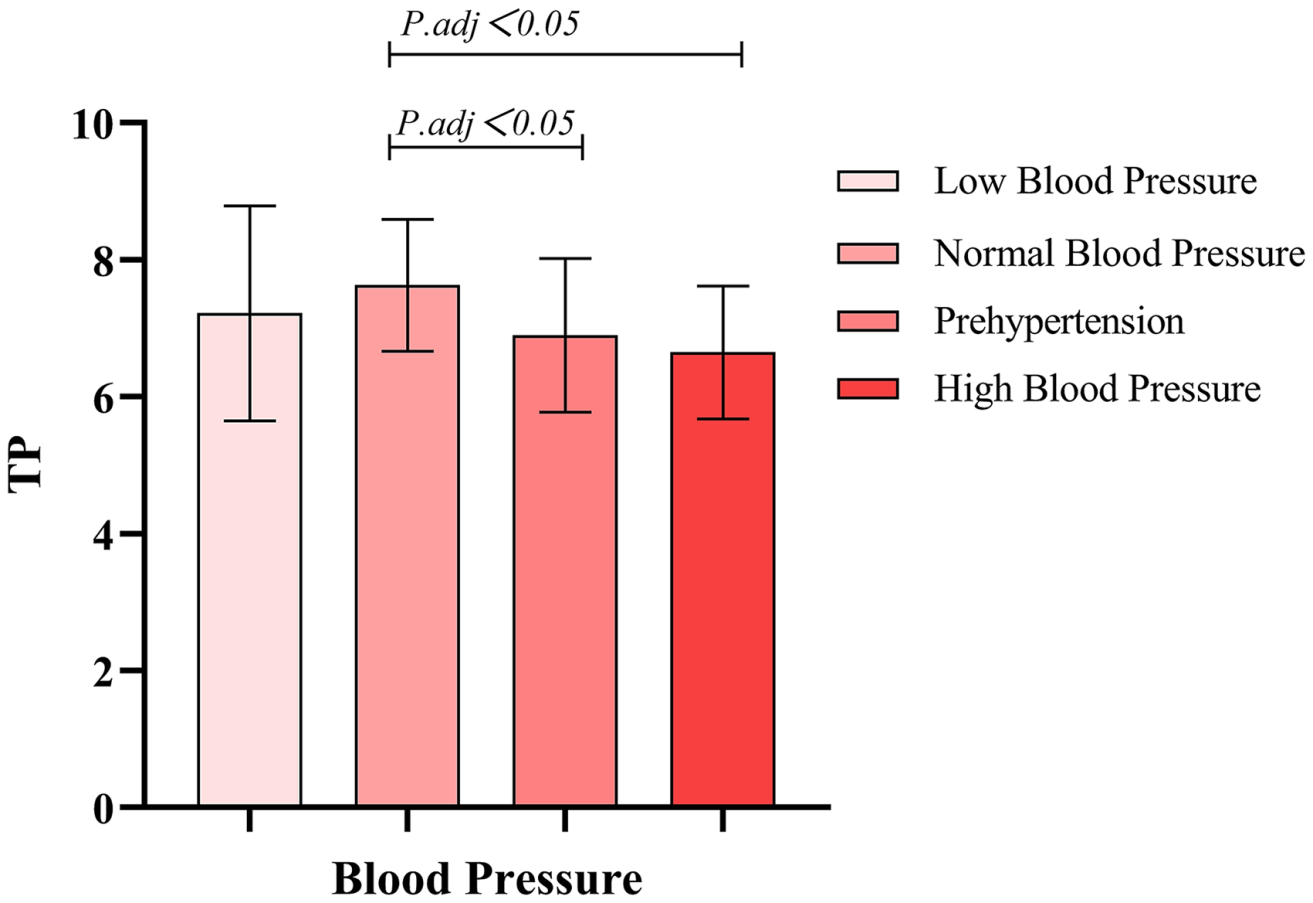
Comparison of different blood pressure levels and TP.

**Figure 2:**
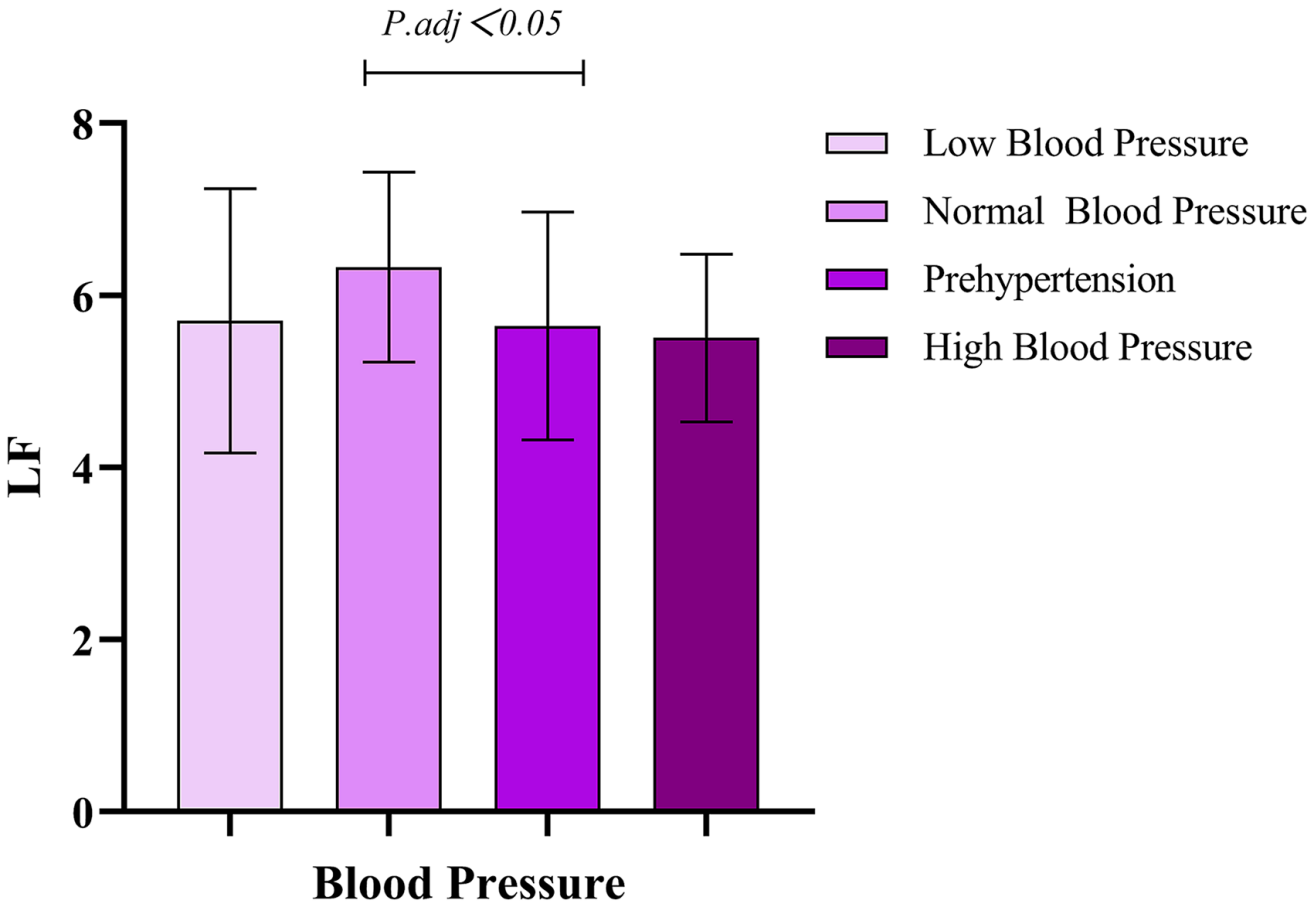
Comparison of different blood pressure levels and LF.

**Figure 3:**
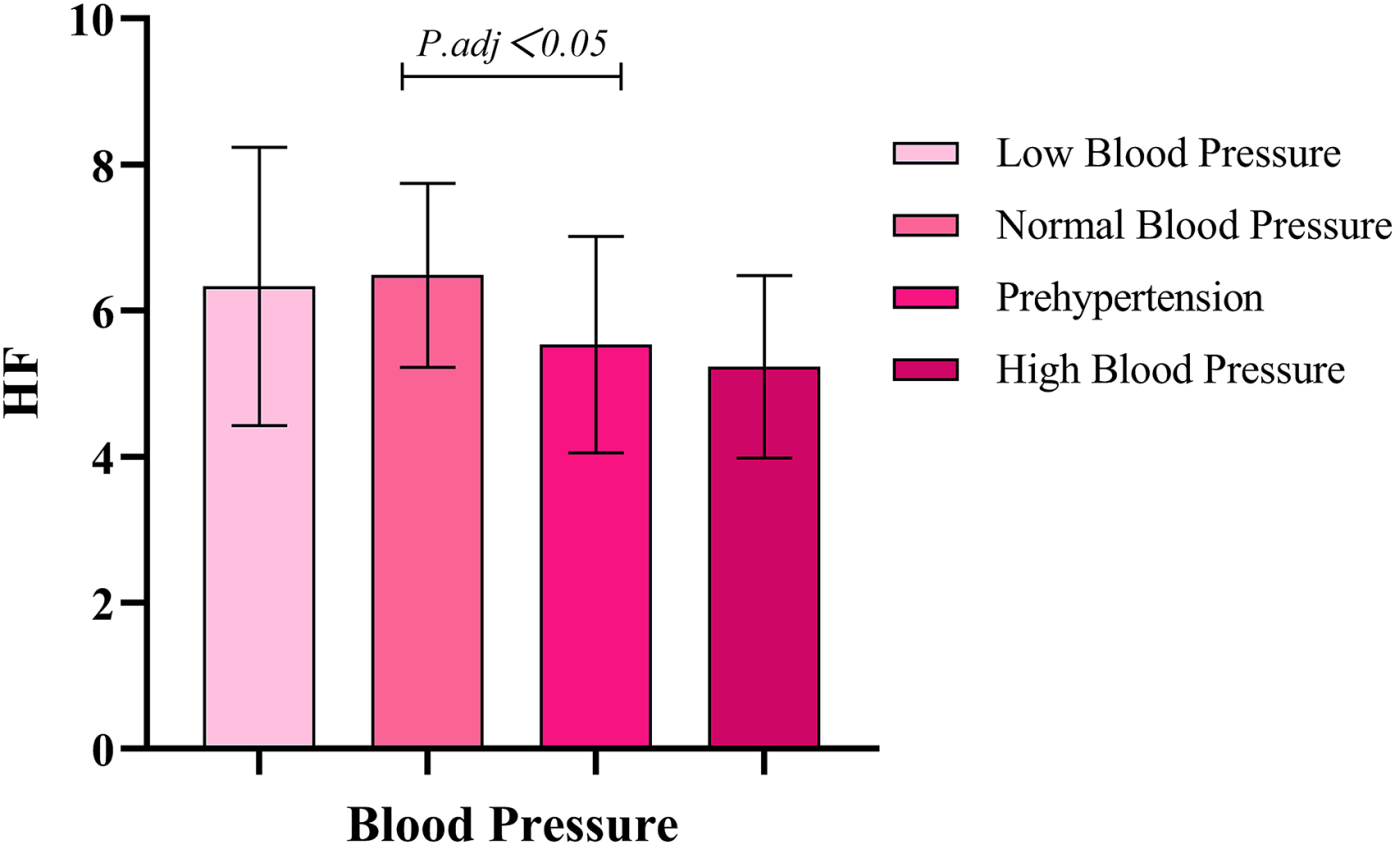
Comparison of different blood pressure levels and HF.

The research results show that there are significant differences between the different blood pressure groups and all HRV indices (*p* < 0.001). Effect size analysis indicates that blood pressure has a moderate-to-large impact on TP and HF (*η*^2^ = 0.118) and a small-to-moderate impact on LF (*η*^2^ = 0.082), as shown in Table 3. Within-group comparisons reveal that, compared with the normal-blood-pressure group, the pre-hypertension and hypertension groups exhibit a significant reduction in HRV, as illustrated in Figures 1–3.

**Table 3:** Comparison of HRV frequency domain indicators for different blood pressure characteristics (^−^*x* ± *s*).

Feature	*n*	TP	LF	HF
Low blood pressure	12	7.22 ± 1.57	5.71 ± 1.54	6.33 ± 1.91
Normal blood pressure	121	7.63 ± 0.96	6.33 ± 1.10	6.49 ± 1.26
Prehypertension	87	6.90 ± 1.12	5.64 ± 1.32	5.54 ± 1.48
High blood pressure	20	6.65 ± 0.57	5.51 ± 0.98	5.24 ± 1.25
*F*		10.47	6.896	10.511
*p*		<0.001	<0.001	<0.001

### 3.2 Single-factor analysis of heart rate variability in different blood pressure groups

#### 3.2.1 Demographic and sociological characteristics

The research results show that there are significant differences in the frequency domain indicators of HRV, such as TP, LF, and HF, among different age groups (*p* < 0.05). Specifically, TP values decrease with age, indicating a reduction in the overall activity level and regulatory capacity of the autonomic nervous system as people age. There are also significant differences in HRV frequency domain indicators between individuals with different marital statuses and educational levels (*p* < 0.05), with unmarried individuals showing better autonomic nervous system regulation compared to married individuals. TP and LF also show significant differences among individuals with different monthly family incomes (*p* < 0.05), while gender does not show significant differences in any HRV indicator (*p* > 0.05), as shown in Table 4.

**Table 4:** Analysis of the impact of demographic and sociological characteristics on heart rate variability (^−^*x* ± *s*).

Item	Feature	*n*	TP	LF	HF
Gender	Male	71	7.29 ± 1.33	6.11 ± 1.47	5.89 ± 1.69
	Female	169	7.25 ± 1.02	5.93 ± 1.14	6.09 ± 1.35
	*t*		0.228	1.043	−0.967
	*p*		0.82	0.298	0.335
Age	18–40 years old	136	7.70 ± 0.92	6.48 ± 1.05	6.53 ± 1.21
	41–65 years old	79	6.74 ± 0.92	5.43 ± 0.99	5.36 ± 1.32
	≥66 years old	25	6.52 ± 1.54	5.03 ± 1.07	5.42 ± 0.22
	*F*		30.681	32.826	22.019
	*p*		<0.001	<0.001	<0.001
Marital status	Unmarried	81	8.01 ± 0.73	6.74 ± 0.93	6.96 ± 1.03
	Married	146	6.91 ± 1.09	5.63 ± 1.19	5.59 ± 1.40
	Divorced	7	6.95 ± 0.40	5.73 ± 0.47	5.55 ± 0.53
	Widowed	6	6.09 ± 1.57	4.52 ± 1.90	4.62 ± 2.38
	*F*		25.172	20.853	22.076
	*p*		<0.001	<0.001	<0.001
Education level	Primary School	10	6.78 ± 1.10	5.54 ± 1.26	5.30 ± 1.07
	Junior High School	25	6.62 ± 1.41	5.20 ± 1.52	5.33 ± 1.85
	High School, Vocational School	79	6.80 ± 0.94	5.50 ± 1.09	5.42 ± 1.25
	College, Bachelor’s Degree	126	7.72 ± 0.96	6.47 ± 1.07	6.61 ± 1.28
	*F*		18.217	16.921	16.845
	*p*		<0.001	<0.001	<0.001
Monthly household income	No Income	4	7.52 ± 0.32	6.21 ± 0.69	6.25 ± 0.27
	Below 1,000 Yuan	14	7.42 ± 1.05	6.29 ± 1.23	6.03 ± 1.53
	Between 1,000 and 3,000 Yuan	70	7.26 ± 1.09	5.96 ± 1.28	5.98 ± 1.31
	Between 3,000 and 5,000 Yuan	98	7.45 ± 1.12	6.18 ± 1.20	6.31 ± 1.49
	More than 5,000 Yuan	54	6.87 ± 1.15	5.55 ± 1.24	5.57 ± 1.53
	*F*		2.601	2.606	2.333
	*p*		0.037	0.037	0.057

#### 3.2.2 Behavioral lifestyle

The research results show that there are significant differences in HRV indicators among individuals with different drinking habits, dietary preferences, and exercise patterns (*p* < 0.05). Specifically, individuals who consume alcohol have higher values of LF and HF compared to those who do not drink alcohol, indicating that alcohol consumers exhibit higher sympathetic and parasympathetic nervous activity than non-drinkers. Smoking and water drinking habits do not show significant differences in HRV indicators (*p* > 0.05), as shown in Table 5.

**Table 5:** Analysis of factors influencing heart rate variability by behavioral lifestyle (^−^*x* ± *s*).

Item	Feature	*n*	TP	LF	HF
Smoking	No	177	7.30 ± 1.13	5.99 ± 1.26	6.11 ± 1.49
	Yes	63	7.16 ± 1.09	5.94 ± 1.22	5.80 ± 1.36
	*t*		0.907	0.303	1.537
	*p*		0.366	0.762	0.127
Drinking	No	171	7.02 ± 1.12	5.73 ± 1.24	5.73 ± 1.47
	Yes	69	7.86 ± 0.87	6.60 ± 1.04	6.78 ± 1.13
	*t*		−5.544	−5.533	−5.37
	*p*		<0.001	<0.001	<0.001
Exercise	Less than once a month	23	7.32 ± 1.30	5.91 ± 1.41	6.20 ± 1.91
	2–3 times a month	62	6.95 ± 1.25	5.64 ± 1.40	5.64 ± 1.56
	2–3 times a week	70	7.15 ± 1.01	5.82 ± 1.11	5.91 ± 1.31
	3–5 times a week	61	7.56 ± 0.96	6.36 ± 1.07	6.34 ± 1.28
	Almost every day	24	7.59 ± 1.06	6.41 ± 1.16	6.42 ± 1.36
	*F*		3.088	3.731	2.482
	*p*		0.017	0.006	0.045
Diet preference	Light taste	121	7.20 ± 1.13	5.94 ± 1.30	5.92 ± 1.49
	Heavy salt	53	7.67 ± 1.03	6.40 ± 1.16	6.59 ± 1.25
	Likes high-calorie food	66	7.05 ± 1.10	5.71 ± 1.13	5.78 ± 1.47
	*F*		5.128	4.77	5.374
	*p*		0.007	0.009	0.005
Drinking water habits	Drinking water on an empty stomach in the morning	62	7.28 ± 1.13	5.99 ± 1.24	6.01 ± 1.54
	No habit of drinking water on an empty stomach, but drinking water in the afternoon	44	7.34 ± 1.02	6.04 ± 1.21	6.20 ± 1.18
	Drinking water irregularly	80	7.39 ± 1.15	6.13 ± 1.26	6.17 ± 1.52
	Only drink water when thirsty”	54	7.00 ± 1.13	5.70 ± 1.24	5.70 ± 1.46
	*F*		1.464	1.331	1.396
	*p*		0.225	0.265	0.245

#### 3.2.3 Physiological indicators

The research results show that there are significant differences (*p* < 0.05) in HRV indicators among patients with diabetes, hyperlipidemia, coronary heart disease, and poor sleep quality. Patients with coronary heart disease have significantly lower values of TP, LF, and HF compared to those without the disease, indicating weaker activity and regulatory capacity of the autonomic nervous system. There is a significant difference between BMI categories and HF (*p* < 0.05), but no significant difference with TP and LF (*p* > 0.05). Specifically, individuals who are obese or overweight have slightly lower parasympathetic activity compared to those with normal body weight, as shown in Table 6.

**Table 6:** Analysis of factors affecting heart rate variability by physiological indicators (^−^*x* ± *s*).

Item	Feature	*n*	TP	LF	HF
BMI classification	Underweight	22	7.84 ± 1.40	6.25 ± 1.47	6.87 ± 1.94
	Normal weight	115	7.17 ± 1.03	5.88 ± 1.13	5.99 ± 1.35
	Overweight	88	7.24 ± 1.12	6.01 ± 1.32	5.91 ± 1.42
	Obese	15	7.28 ± 1.16	6.17 ± 1.32	5.77 ± 1.42
	*F*		2.288	0.745	2.839
	*p*		0.079	0.526	0.039
Diabetes	No	216	7.33 ± 1.09	6.04 ± 1.22	6.12 ± 1.42
	Yes	24	6.65 ± 1.25	5.44 ± 1.33	5.19 ± 1.58
	*t*		2.538	2.106	2.776
	*p*		0.017	0.044	0.01
Hyperlipidemia	No	158	6.81 ± 0.95	5.39 ± 0.98	5.51 ± 1.32
	Yes	82	8.13 ± 0.88	7.11 ± 0.87	7.03 ± 1.17
	*t*		−10.755	−13.942	−9.129
	*p*		<0.001	<0.001	<0.001
Coronary heart disease	No	218	7.37 ± 1.09	6.08 ± 1.23	6.15 ± 1.42
	Yes	22	6.24 ± 0.92	4.97 ± 0.94	4.87 ± 1.33
	*t*		5.381	4.107	4.278
	*p*		<0.001	<0.001	<0.001
Sleep quality	Poor	106	7.03 ± 1.22	5.75 ± 1.34	5.76 ± 1.59
	Good	134	7.45 ± 1.00	6.16 ± 1.34	6.24 ± 1.32
	*t*		−2.968	−2.591	−2.509
	*p*		0.003	0.01	0.013

#### 3.2.4 Physiological factors

Research results show that anxiety, cognitive impairment, and various HRV indicators have significant differences (*p* < 0.05). Specifically, individuals with anxiety states have higher values of LF and HF, indicating increased sympathetic and parasympathetic nervous activity, leading to an imbalance in autonomic nervous system regulation. There is no significant difference between depression and HRV indicators (*p* > 0.05), as shown in Table 7.

**Table 7:** Analysis of factors influencing heart rate variability by psychological factors (^−^*x* ± *s*).

Item	Feature	*n*	TP	LF	HF
Depression	No	233	7.27 ± 1.12	5.99 ± 1.24	6.04 ± 1.47
	Yes	7	6.89 ± 1.11	5.67 ± 1.56	5.55 ± 1.20
	*t*		0.895	0.546	1.057
	*p*		0.404	0.604	0.328
Anxiety	No	105	7.03 ± 1.22	5.75 ± 1.34	5.56 ± 1.59
	Yes	135	7.45 ± 1.00	6.16 ± 1.14	6.24 ± 1.31
	*t*		−2.941	−2.478	−2.52
	*p*		0.004	0.014	0.013
Cognitive impairment	No memory problems	203	7.40 ± 1.02	6.14 ± 1.15	6.17 ± 1.35
	Possible memory issues	37	6.50 ± 1.31	5.13 ± 1.40	5.25 ± 1.78
	*t*		3.951	4.141	3.01
	*p*		<0.001	<0.001	0.004

### 3.3 Impact factors of heart rate variability in different blood pressure groups

#### 3.3.1 Variable importance ranking

**Figure 4:**
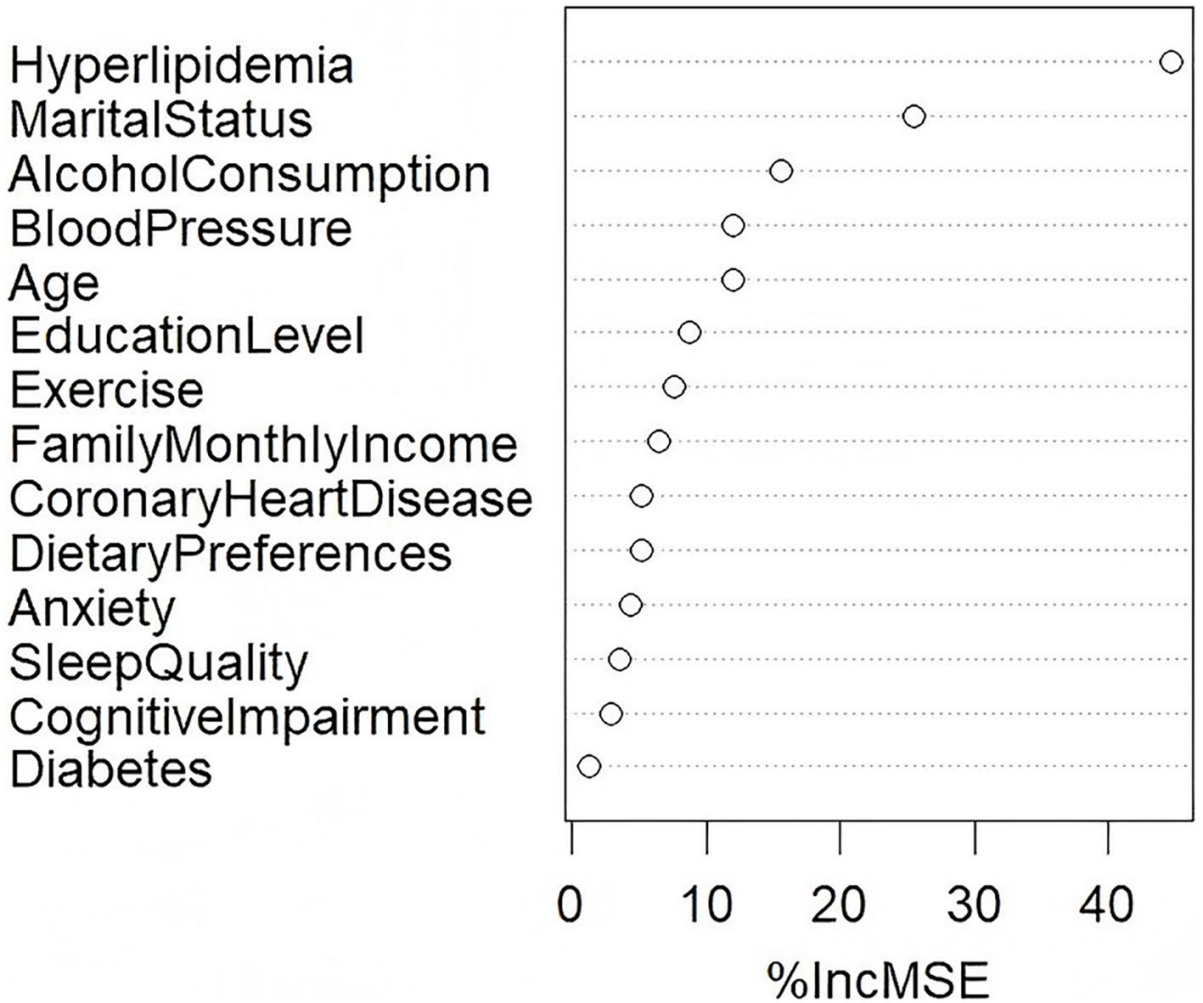
Importance ranking of TP influencing factors.

Taking the various frequency domain indicators of HRV (TP, LF, and HF) as dependent variables, and the factors that significantly influenced HRV in the univariate analysis of different blood pressure groups as independent variables, a random forest model was constructed using the Random Forest package in R studio software. 20% of the samples were used as the test set, and 80% as the training set. The Importance function was applied to rank the variables, where %Inc. MSE represents the increase in mean squared error (Mean Squared Error). The larger the %Inc. MSE value, the greater the importance of the variable in the influencing factors (16). The variable assignment situation of the random forest model is shown in Table 8. The results showed that the highest importance variables for TP, LF and HF were all high lipid levels, as detailed in Figure 4.

**Table 8:** Variable assignment of random forest model for HRV influencing factors.

Related variables		Assignment
Age	X1	18–40 years = 1,41–65 years = 2,≥66 years = 3
Marital status	X2	Unmarried = 1, Married = 2, Divorced = 3, Widowed = 4
Education level	X3	Primary school = 1, Junior high school = 2, High school, technical school = 3, College, undergraduate = 4
Monthly household income	X4	No income = 1, Below 1,000 yuan = 2,1,000–3,000 yuan = 3,3,000–5,000 yuan = 4, Above 5,000 yuan = 5
Drinking	X5	No = 0, Yes = 1
Exercise	X6	Less than once a month = 1, 2–3 times a month = 2, 1–2 times a week = 3, 3–5 times a week = 4, About once a day = 5
Dietary preference	X7	Light taste = 1, Heavy salt intake = 2, Likes high-calorie foods = 3
Blood pressure	X8	Low blood pressure = 1, Normal blood pressure = 2, Prehypertension = 3, High blood pressure = 4
Diabetes	X9	No = 0, Yes = 1
Hyperlipidemia	X10	No = 0, Yes = 1
Coronary heart disease	X11	No = 0, Yes = 1
BMI classification	X12	Underweight = 1, Normal weight = 2, Overweight = 3, Obese = 4
Sleep quality	X13	Poor = 1, Good = 2
Anxiety	X14	No = 0, Yes = 1
Cognitive impairment	X15	No memory problems = 0, Possible memory problems = 1

#### 3.3.2 Variable selection

**Figure 5:**
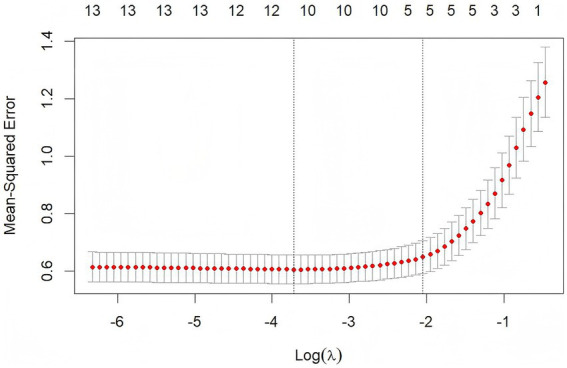
Feature variable screening based on LASSO regression – TP.

**Figure 6:**
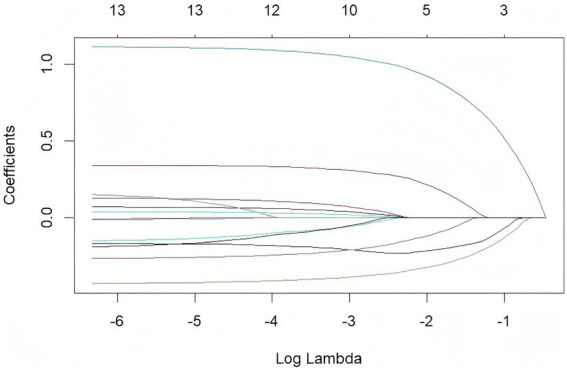
Lasso regression analysis variable interpretation plot – TP.

Based on the results of Figure 4, the glment function in R Studio was used to incorporate variables that showed significant differences in the univariate HRV analysis into LASSO regression models, as illustrated in Figure 5. The left and right vertical dashed lines represent lambda.min (the penalty coefficient *λ* corresponding to the minimum cross-validation error) and lambda.1se (the penalty coefficient *λ* corresponding to the minimum cross-validation error plus one standard error), respectively. Mean-squared error (MSE) is a core metric for measuring the discrepancy between a model’s predictions and the true values; log(*λ*) denotes the natural logarithm of the penalty coefficient λ. Within the [lambda.min, lambda.1se] interval, the model’s error variation is minimal (15). When λ equals 0.02419 (the left dashed line, lambda.min), the TP model achieves the smallest error, with 10 variables selected for TP. When λ equals 0.01973, the LF model attains the smallest error, also with 10 variables selected for LF. When λ equals 0.03348, the HF model reaches the smallest error, with 12 variables selected for HF. These selected variables were then entered into multivariate linear regression models (see Figures 5, 6 for details).

### 3.4 Multifactorial analysis of heart rate variability in different blood pressure populations

To develop the final predictive model, we performed a multiple stepwise linear regression analysis using IBM SPSS Statistics software. All effective variables identified by the LASSO regression were entered as candidate independent variables. In the regression dialog box, the method was set to “Stepwise.” SPSS’s stepwise regression automatically selects variables based on the *p*-value of the partial F-test, with the default entry criterion of *p* ≤ 0.05 and removal criterion of *p* ≥ 0.10, which aligns with the objectives of this study. Ultimately, only those variables that remained significant (*p* < 0.05) after adjusting for the other variables were retained in the final multiple linear regression model. The results indicated that factors such as age, marital status, alcohol consumption, exercise, blood pressure, and hyperlipidemia all affect autonomic nervous system balance in this population, as shown in Table 9.

**Table 9:** Multivariate linear regression analysis of factors affecting heart rate variability.

Dependent variable	Independent variable	*β*	SE	Beta	*t*	*p*	VIF
TP	Constant	8.566	0.214		40.02	<0.001	
	Hyperlipidemia	1.152	0.104	0.489	11.075	<0.001	1.042
	Marital status	−0.431	0.093	−0.244	−4.654	<0.001	1.466
	Drinking	0.407	0.113	0.165	3.599	<0.001	1.124
	Blood pressure	−0.256	0.07	0.164	−3.636	<0.001	1.093
	Age	−0.279	0.089	−0.169	−3.142	0.002	1.544
LF	Constant	6.786	0.239		28.383	<0.001	
	Hyperlipidemia	1.537	0.104	0.587	14.719	<0.001	1.043
	Age	−0.425	0.089	−0.231	−4.751	<0.001	1.551
	Marital status	−0.382	0.093	−0.194	−4.113	<0.001	1.466
	Drinking	0.252	0.117	0.092	2.144	0.033	1.201
	Exercise	0.119	0.045	0.109	2.653	0.009	1.110
	Blood pressure	−0.179	0.071	−0.103	−2.504	0.013	1.118
HF	Constant	7.678	0.298		25.742	<0.001	
	Hyperlipidemia	1.3	0.148	0.423	8.755	<0.001	1.041
	Marital status	−0.707	0.114	−0.307	−6.219	<0.001	1.090
	Blood pressure	−0.423	0.100	−0.209	−4.247	<0.001	1.080
	Drinking	0.654	0.156	0.203	4.202	<0.001	1.045

## 4 Discussion

According to the results of demographic studies and HRV research, the frequency domain indicators of HRV decrease with age, indicating that the overall activity level and regulatory capacity of the autonomic nervous system weaken as people age. This is consistent with the findings of Wang (14), who studied 483 civil servants in a direct-controlled organ in Changchun. The results showed that TP, LF, and HF values decreased with age, indicating that the regulatory capacity of the autonomic nervous system weakens as people age. Jiang et al. (17) pointed out that age becomes one of the risk factors for autonomic nervous dysfunction. As age increases, the body’s autonomic nervous control function degenerates, leading to a shift in the balance between the sympathetic and parasympathetic nerves toward the sympathetic side. Wu (18) also showed that various indicators of HRV decrease with age, indicating that the autonomic nervous system is affected and overall health declines. Marital status is another factor affecting HRV. Unmarried individuals have higher values of TP, LF, and HF compared to married or divorced individuals, indicating that unmarried individuals have better autonomic nervous regulation ability.

Research results on lifestyle behaviors and HRV show that alcohol consumption has a significant impact on HRV. Karpyak et al. (19) found that high alcohol intake is associated with a decrease in HRV. Chen et al. (20) discovered that HRV is influenced by physical activity, and the intensity of physical activity is positively correlated with the sympathetic nervous system. Fu et al. (21) analyzed the effects of Tai Chi and fitness exercises, both moderate-intensity physical activities, on HRV in patients with primary hypertension. The results showed that these two exercises can effectively improve the autonomic nervous function of patients with primary hypertension.

The physiological indicators and HRV research results show that blood pressure has a significant impact on HRV. Jarczok et al. (22) found through a health assessment of 9,550 working adults that HRV may be associated with an increased risk of cardiovascular events. This study confirmed for the first time the correlation between reduced HRV and elevated cardiovascular risk, suggesting that HRV could potentially become a simple and novel indicator for assessing cardiovascular risk (23). The research results of Fang et al. (24) indicate that a decrease in HRV indicators is significantly associated with an increased risk of cardiovascular events. Yue et al. (25) found that both patients with masked hypertension and those with primary hypertension had significantly lower HRV parameters compared to the normal population, but there was no significant difference between the two groups. Virtanen et al. (6) studied and found that all absolute measurements of HRV in hypertensive patients were relatively lower compared to the healthy population. Zhao (26) found in a study on HRV and prehypertension that individuals with excessive sympathetic tone had a higher detection rate of prehypertension than those with normal tone, and elevated HRV significantly increased the risk of developing prehypertension. Moreover, related clinical research has shown that the sympathetic nervous system can promote angiogenesis and tumor progression in various cancers (27).

Furthermore, this study has several limitations. First, the sample was sourced from a single urban center, which limits the external validity of the findings when generalizing to other populations. Second, the sample size was relatively limited, particularly within the hypotension and hypertension subgroups, which may affect the statistical power of complex multivariate models and increase the risk of Type II errors. Therefore, analyses focusing on these subgroups should be considered exploratory. Finally, although we included various demographic and behavioral factors in our analysis, we did not comprehensively measure and control for known confounders affecting HRV, such as detailed caffeine intake and specific medication history, which might have influenced the precision of the results. Despite these limitations, this study, utilizing modern statistical methods like Random Forest and LASSO regression on a systematically quality-controlled community sample, identified hyperlipidemia as the most significant factor influencing HRV, with other notable factors including blood pressure, marital status, alcohol consumption, age, and physical exercise. These findings have important public health implications. Based on the above findings, we recommend the following measures for the community population to maintain autonomic nervous system health: advocate for regular physical exercise and develop personalized exercise plans; strengthen the management of blood pressure and blood lipids, reduce the intake of high-calorie and fried foods, and control alcohol consumption; simultaneously, promote public awareness and knowledge regarding the maintenance of autonomic nervous system health. Future studies should expand the sample size, include more blood pressure abnormality subgroups, and conduct long-term follow-up to clarify the causal relationship between HRV and cardiovascular events, thereby providing stronger evidence for the early prevention of cardiovascular diseases.

## Data Availability

The original contributions presented in the study are included in the article/supplementary material, further inquiries can be directed to the corresponding author.
